# Monitoring Monkeypox: Safeguarding Global Health through Rapid Response and Global Surveillance

**DOI:** 10.3390/pathogens12091153

**Published:** 2023-09-11

**Authors:** Marta Giovanetti, Eleonora Cella, Sonia Moretti, Fabio Scarpa, Alessandra Ciccozzi, Svetoslav Nanev Slavov, Francesca Benedetti, Davide Zella, Giancarlo Ceccarelli, Massimo Ciccozzi, Alessandra Borsetti

**Affiliations:** 1 Instituto Rene Rachou Fundação Oswaldo Cruz, Belo Horizonte 30190-009, Minas Gerais, Brazil; 2 Sciences and Technologies for Sustainable Development and One Health, University Campus Bio-Medico of Rome, 00128 Rome, Italy; 3 Climate Amplified Diseases and Epidemics (CLIMADE), Brazil; 4 Burnett School of Biomedical Sciences, University of Central Florida, Orlando, FL 32816, USA; eleonora.cella@yahoo.it; 5 National HIV/AIDS Research Center (CNAIDS), National Institute of Health, 00161 Rome, Italy; sonia.moretti@iss.it; 6 Department of Biomedical Sciences, University of Sassari, 07100 Sassari, Italy; fscarpa@uniss.it; 7 Unit of Medical Statistics and Molecular Epidemiology, University Campus Bio-Medico of Rome, 00128 Rome, Italy; ale_ciccozzi97@icloud.com (A.C.); m.ciccozzi@unicampus.it (M.C.); 8 Butantan Institute, Blood Center of Ribeirão Preto, Faculty of Medicine of Ribeirão Preto, University of São Paulo, Ribeirão Preto 05508-220, São Paulo, Brazil; svetoslav.slavov@hemocentro.fmrp.usp.br; 9 Department of Biochemistry and Molecular Biology, Institute of Human Virology and Global Virus Network Center, University of Maryland School of Medicine, Baltimore, MD 21201, USA; fbenedetti@ihv.umaryland.edu (F.B.); dzella@ihv.umaryland.edu (D.Z.); 10 Department of Public Health and Infectious Diseases, Sapienza University of Rome, 00161 Rome, Italy; giancarlo.ceccarelli@uniroma1.it

**Keywords:** monkeypox, genomic surveillance, zoonotic disease

## Abstract

Monkeypox, a viral zoonotic disease, has emerged as a significant global threat in recent years. This review focuses on the importance of global monitoring and rapid response to monkeypox outbreaks. The unpredictable nature of monkeypox transmissions, its potential for human-to-human spread, and its high morbidity rate underscore the necessity for proactive surveillance systems. By analyzing the existing literature, including recent outbreaks, this review highlights the critical role of global surveillance in detecting, containing, and preventing the further spread of monkeypox. It also emphasizes the need for enhanced international collaboration, data sharing, and real-time information exchange to effectively respond to monkeypox outbreaks as a global health concern. Furthermore, this review discusses the challenges and opportunities of implementing robust surveillance strategies, including the use of advanced diagnostic tools and technologies. Ultimately, these findings underscore the urgency of establishing a comprehensive global monitoring framework for monkeypox, enabling early detection, prompt response, and effective control measures to protect public health worldwide.

## 1. Introduction

The significance of the impact of global environmental changes in the emergence of zoonotic diseases has grown considerably. Human actions, including deforestation, land clearance, and a rise in tourism, have led to disruptions in ecosystems. These disruptions have, in turn, increased the chances of closer interactions between humans, animals, and their vectors [1]. Among these interactions, those involving non-human primates (NHPs) hold particular importance due to their genetic and behavioral resemblances to humans. These interactions also include direct transmission through vectors, contact with contaminated materials, and other pathways that contribute to the spread of zoonotic pathogens. It is estimated that around 25% of emerging infectious diseases in humans originate from primate host species, underscoring the significance of understanding and monitoring zoonotic transmission [2]. Zoonosis, as defined by the World Health Organization (WHO), refers to a disease naturally transmitted from animals to humans. Animals play a critical role in the maintenance of these infections within nature in the form of zoonotic cycles. Zoonotic transmission is believed to be the primary pathway through which infectious agents are introduced to human populations [3]. Human-driven activities, such as deforestation, land clearance for agriculture, and increased tourism, have disrupted ecosystems and habitats that have remained relatively unchanged for centuries. Within these ecosystems, non-human and human primates may coexist with humans and harbor pathogens that are capable of being transmitted to and infecting humans. The genetic and behavioral similarities between humans and NHPs make the latter a potential source of clinical infections that could lead to pandemics. RNA viruses, known for their high nucleotide substitution rate, have a greater adaptability to new hosts, increasing the likelihood of interspecies transmission. However, successful cross-species transmission does not guarantee an efficient spread within the new population. NHPs, human primates, are our closest relatives, making them a potential source of infections and pandemics.

Transmission from monkeys to humans can occur through exposure to sick or dead animals, keeping them as pets, or through hunting, slaughtering, and subsequent dismembering. Another way new infectious agents can emerge is through the interaction and recombination of two distinct agents, resulting in new properties with their potential for pathogenicity, infectivity, or transmissibility among humans [3]. The recent outbreak of monkeypox reported in multiple countries highlights the risk of emerging animal-sourced viruses with epidemic or pandemic potential, posing a significant threat to global public health [4]. Understanding zoonotic transmission and monitoring outbreaks among wildlife populations is crucial. It is imperative to identify and characterize animal viruses with the highest risk of spillover to humans. This review aims to briefly discuss monkeypox, a virus transmitted from monkeys to humans, which has the potential to cause disease in both humans and other species, potentially leading to the emergence of new diseases or epidemics.

## 2. Epidemiology of Monkeypox and 2023 Reemergence

**Figure 1:**
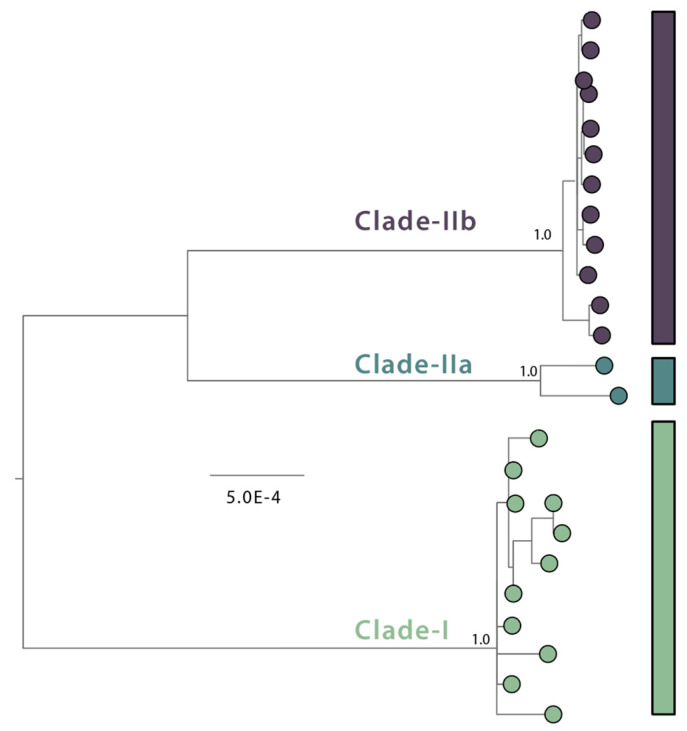
MPXV nomenclature. Midpoint-rooted maximum likelihood (ML) phylogenetic tree with n = 25 MPXV whole genome sequences from distinct MPXV genotypes (Clade I, n = 11; Clade IIa, n = 2A; Clade IIb, n = 12). The three distinct MPXV clades are indicated, representing the deep diversity of MPXV. Clade I correspond to the prior “Congo Basin clade”, while Clades IIa and IIb correspond to the prior “West African clade”. Clade IIb contains a group of genomes from 2017, 2018 and 2022–2023 sampled from human cases that likely represent sustained human-to-human transmission. Different clades are represented by distinct colors.

Monkeypox is a Poxviridae family virus that has enclosed double-stranded DNA and belongs to the Orthopoxvirus genus. This virus was discovered in humans in the Democratic Republic of the Congo (DRC) in 1970 and has subsequently been detected in African countries and elsewhere. Notable outbreaks occurred in the Democratic Republic of the Congo in 1996–1997 and in the United States in 2003, both connected to contact with infected pet prairie dogs. An atypical monkeypox epidemic was observed throughout numerous Member States in May 2022, prompting the WHO to declare it a Public Health Emergency of International Concern [5,6,7,8,9]. However, coordinated efforts by governments and impacted communities, particularly men who had sex with men (MSM), have helped to keep the outbreak under control in the months since and until 2023. Infection with monkeypox has also been reported in people moving from Nigeria to other nations. The virus spreads by a variety of methods, including direct contact with infected animals, meat-eating, and human-to-human contact, particularly through contagious skin or other lesions, including those in the mouth or on the genitals. The incubation period of infection is 1–2 weeks, and onset usually occurs 1 to 21 days following exposure [8]. Fever, muscle discomfort, headache, exhaustion, and respiratory problems are common early symptoms of monkeypox infection. The typical rash develops 1–10 days after exposure and continues through numerous stages, such as macular, papular, vesicular, and pustular. Lesions can occur on any area of the body and progress from coalesce into bullae and bleed. While most cases are self-limiting, severe cases can occur, particularly in susceptible populations who may have symptoms for a prolonged period of time. Monkeypox symptoms are comparable to those of other members of this family, such as smallpox and chickenpox, and include fever, weakness, headache, and lymphadenopathy (Table 1). Monkeypox is divided into two taxonomic groups: the central African (previously Congo Basin) clades, now known as Clade I, and the old West African clades, now known as Clade II (Figure 1) [5].

Clade I is associated with more severe disease and increased transmissibility, with a case/fatality ratio (CFR) larger than 10%, whereas Clade II has a CFR of less than 1% [6,7]. Rodents and non-human primates are examples of animal hosts [8].

Clade II was discovered during the 2017–2019 outbreaks in Nigeria, Singapore, the United Kingdom, and the United States, and it is accountable for the rapid global expansion seen in 2022 [10,11,12]. Several works of research comprising the study of 337 new monkeypox genomes published in 2022 offer light on the different features and molecular compositions of this new clade. Luma et al. [13] validated its separation into three distinct lineages, identifying the one that appeared in Europe between the end of 2021 and the beginning of 2022 as B.1 and blaming it for the majority of infections [13]. Happi et al. [11] recently advocated designating the new clade (the third clade) Clade IIb and Clade II Clade IIa, as they both descend from the same former West African clade. Clade II (previously known as the West African clade) now includes Clades IIa and IIb, according to this classification.

The present reemergence, as observed in the 2022 outbreak, is related to a sub-clade IIb lineage, namely lineage B.1. More than 7000 genomes of lineage B.1 are already available in GISAID [14], and when paired with its progeny, it represents the most common monkeypox lineage worldwide. The sequencing of the B.1 clade has revealed a large number of single-nucleotide polymorphisms, indicating possible improvements in inter-human transmission, mutation, and adaption capabilities [15,16]. Several scholars have recently noticed a considerable rise in the rate of evolution within the B.1 lineage (see, for example, [17]). In fact, lineage B.1 evolves at a higher rate than others, with roughly 10–4 substitutions per site each year [18]. In any case, a recent study indicated that the human monkeypox virus (MPXV) demonstrates rapid evolution, which can be impacted by APOBEC3 activity [15]. In general, the multi-country outbreak 22–23 followed a pattern similar to that seen in seasonal flu outbreaks [16,19].

In this regard, the multi-country monkeypox outbreak should not be underestimated, and constant molecular monitoring at the genome level is required to obtain a comprehensive and detailed understanding of each cluster and geographic pattern. Early discovery, swift reaction, and cross-border coordination are critical in reducing the effects of future monkeypox epidemics. Monitoring genetic changes and following the spread of these clades are critical steps in preparing for the increasing threats posed by monkeypox and protecting world health. The severity of this disease is determined by the patient’s immune condition, route of exposure, and viral strain. West African monkeypox has a milder clinical course and minimal human-to-human transmission, but Central African monkeypox is more severe and has documented person-to-person transmission. There are no licensed treatments for monkeypox at the moment; however, two medicines have proven success in animal models. Although the smallpox vaccine has been beneficial in preventing monkeypox, the termination of a global smallpox vaccination has increased susceptibility, particularly among younger populations [20]. Despite the fact that vaccination and antiviral medication were approved in 2019 and 2022, respectively, limited access remains a serious barrier. Ongoing research is attempting to develop a monkeypox vaccine that can overcome limitations and give targeted protection. Various approved tests can be used to diagnose monkeypox, while global efforts are required to improve monitoring, develop viable vaccinations, and increase treatment accessibility, ultimately contributing to the control and prevention of monkeypox outbreaks.

## 3. Challenges in Detecting and Responding to Monkeypox

Accurate and prompt diagnosis is critical for controlling monkeypox outbreaks. Molecular assays, such as the polymerase chain reaction (PCR), are critical for finding the virus’s genetic material in patient samples. However, due to genetic similarities with other orthopoxviruses, finding unique PCR-based diagnostics for monkeypox can be difficult [20]. In impacted areas, the limited availability and accessibility of PCR equipment and expertise may further impede the molecular testing of this virus. Serological testing is critical for verifying instances of monkeypox and determining previous infections. To detect monkeypox-specific antibodies, ELISAs and neutralization tests are routinely utilized (Table 2). However, complications arise due to cross-reactivity with other orthopoxviruses and the possibility of false–positive results, particularly in people who have already been immunized against smallpox [20]. A priority is the development of highly specific serological techniques to distinguish monkeypox antibodies from similar viruses [9]. Serological approaches that have been improved can reliably identify the disease load and guide public health responses during outbreaks [8].

### Diagnostic Assays: Direct and Indirect Methods for the MPXV Screening

Because of their speed, sensitivity, and specificity, nucleic acid amplification tests (NAATs) are the preferred approach for viral detection and characterization. NAAT forms have rapidly evolved over the last two decades, giving numerous methodologies such as conventional PCR, nested PCR, and real-time PCR, each with advantages and limitations (Table 2). During earlier monkeypox epidemics, several diagnostic techniques were well-established [21]. The WHO has recommended quantitative real-time PCR (RT-qPCR) alone or in combination with sequencing [21].

Among the range of techniques evaluated rigorously in both laboratory and field settings to ascertain their precision and reliability, the following stand out: 1. Real-time PCR has been established as the gold standard for monkeypox viral diagnosis. It outperforms other procedures due to its speed, sensitivity, specificity, and high throughput. By targeting specific genes such as DNA polymerase and envelope protein, real-time PCR can not only identify the presence of the virus but also measure the viral load and analyze the course of infection [22].

1. Multiplex real-time PCR techniques have been developed to detect multiple targets at the same time, such as monkeypox and varicella-zoster. For viral detection, these assays use particular gene sections for each virus and distinct dye/quencher combinations. They have a high specificity and sensitivity, detecting the presence of the targeted viruses [23].
2. The pan-orthopoxvirus PCR/ESI-MS approach leverages the T5000 platform to identify all orthopoxviruses in a single reaction. This approach provides fast and precise identification without the use of sequencing [23].
3. Loop-mediated isothermal amplification (LAMP) is a high-sensitivity and specificity nucleic acid amplification method. LAMP can detect monkeypox and distinguish between virus strains. It is simple to implement and can be used in resource-limited settings [24].
4. Recombinase polymerase amplification (RPA) is another isothermal approach that allows for lower-temperature DNA amplification. It has a quick diagnosis, does not require complicated equipment, and is stable in a variety of environmental situations. RPA can be beneficial, particularly in areas with limited access to molecular techniques [25].
5. GeneXpert is a portable technology that integrates sample preparation, real-time PCR, and detection into a single closed system. It has advantages, such as requiring fewer samples, being simple to use, and being able to detect several infectious agents, allowing for the effective management and surveillance of monkeypox infections [26].
6. Although immunohistochemistry and electron microscopy can provide useful information on virus–host interactions and tissue involvement, they are not commonly used in clinical settings [27].
7. Cell culture techniques are appropriate for virus biology and pathobiology research, but they are not suggested for routine diagnostic use [28].
8. Serological methods such as ELISA can quantify exposure and vaccine efficacy, although they have limitations in distinguishing orthopoxvirus species [29].
9. Although rapid antigen tests and lateral flow assays provide quick and easy diagnosis in minutes, their sensitivity and false–positive rates should be evaluated [30].
10. To evaluate the features and diversity of monkeypox viruses, genomic sequencing using either Sanger or next-generation sequencing technologies is advised. The World Health Organization strongly encourages the sharing of genetic data through databases [31].

These direct and indirect procedures and techniques are critical for the detection, characterization, and surveillance of the monkeypox virus. They contribute to its control and prevention by providing rapid and precise identification, studying virus–host interactions, and evaluating vaccination efficacy. Continued advances in molecular technologies could improve our ability to protect world health by responding quickly to monkeypox and other infectious illnesses and monitoring them globally.

## 4. Importance of Rapid Response and Global Surveillance: Enhancing Monkeypox Surveillance and Response

Rapid responses and worldwide surveillance are critical components of improving viral surveillance and response efforts. The significance of a rapid and coordinated response to monkeypox epidemics cannot be emphasized. With the re-emergence of monkeypox, a global response was quickly initiated, resulting in the implementation of epidemiological and clinical studies, new serological tools, vaccine clinical trials, genomic analyses, and wastewater surveillance based on the knowledge and lessons learned from the SARS-CoV-2 pandemic [32].

Given the possibility that the circulating monkeypox virus adapts to the human host, it is critical to continuously monitor its genetic modifications. This monitoring is required to anticipate rapid epidemiological shifts and successfully prevent the possibility of a pandemic [33]. The continuous sequencing of entire monkeypox virus genomes is crucial for detecting not just single nucleotide polymorphisms but also intragenic frameshifts or premature stop codons. These genomic changes can provide an early warning sign of probable gene loss, which is critical for comprehensive monitoring [33].

The following are the primary essential points for effective surveillance and response:

- Early Detention: by detecting and confirming cases as soon as possible, public health authorities can undertake the necessary actions to prevent this virus from spreading further.
- Timely Public Health Interventions: a quick response allows for the adoption of time-sensitive public health interventions, such as case isolation, contact tracing, and vaccination programs.
- Outbreak Containment: the timely sharing of surveillance data and information enables a coordinated response.
- Identifying High-Risk locations: global surveillance initiatives aid in the identification of high-risk locations where monkeypox transmission is more likely to occur or emerge.
- Viral Evolution Monitoring: the continuous surveillance and sequencing of monkeypox virus genomes can provide vital insights into viral evolution and genomic alterations.
- International Collaboration: global surveillance networks enable countries and organizations to collaborate and share information. The coordination of activities can result in a more complete and rapid response, resulting in improved disease control and prevention.

A joint approach is useful for enhancing the scientific community’s efforts in monitoring and controlling the spread of monkeypox. The response to this outbreak can be reinforced at the local, regional, and global levels by utilizing the strengths of both clinical and environmental laboratory surveillance [34]. The non-invasive, cost-effective, and efficient nature of wastewater surveillance in tracking trends at the community level has increased its appeal [35]. Wastewater surveillance, which involves monitoring untreated wastewater, is critical in identifying emerging infections within populations and estimating their regional and temporal distribution, as well as the circulation of various pathogen types [35]. It is possible to overcome and augment certain shortcomings associated with the clinical monitoring strategy by using a combination approach, such as including wastewater-based surveillance. These limitations include delayed discovery, underreporting, a focus on individuals, and reliance on symptomatic cases. 

There are several resources available to assist the scientific community in quickly accessing monkeypox genomic information, including the Monkeypox Virus Resource (MPoxVR; https://ngdc.cncb.ac.cn/gwh/poxvirus/) [36] and GISAID [14], on a dedicated page, NCBI (https://www.ncbi.nlm.nih.gov/labs/virus/vssi/ Furthermore, a surveillance dashboard, MpoxRadar (https://mpoxradar.net/), has been implemented to readily examine genetic data [37].

## 5. Future Directions and Recommendations

In a constantly changing environment, infectious illnesses such as monkeypox develop and reappear, posing serious hazards to global health [38]. In monitoring and controlling monkeypox epidemics, the requirement for a prompt response and effective surveillance methods becomes critical. Improving worldwide surveillance is not only critical but also required for early detection and response to these epidemics [39]. It requires governments and international organizations to cooperate together to build robust monitoring networks and establish methods for reporting cases, sharing data, and confirming diagnoses in laboratories [39]. Integrating monkeypox surveillance into current disease monitoring platforms could improve their overall readiness and response. However, detection alone is insufficient. Rapid and accurate diagnostic technologies are critical for timely case identification and epidemic control. Investment in the development of point-of-care diagnostic assays for monkeypox is critical [40]. These tests, such as lateral flow assays or handheld PCR equipment, should not only be rapid and easy to use in resource-constrained settings but also user-friendly, affordable, and capable of properly identifying monkeypox from other infections. To combat monkeypox effectively, a better understanding of its transmission dynamics is required. More research is needed to understand the mechanics of human-to-human transmission, identify potential reservoirs and vectors, and assess the impact of environmental factors on disease transmission. By undertaking such studies, targeted interventions, epidemic response tactics, and preventive measures can be improved to tackle this infectious danger [40]. While the smallpox vaccination has demonstrated some success in preventing monkeypox, its limitations necessitate the development of a separate monkeypox vaccine. The focus of research should be on developing safe and effective vaccinations that provide long-term immunity against monkeypox. Investigating novel vaccination techniques, such as viral-vectored vaccines or DNA-based vaccines, could provide new avenues for immunizing vulnerable populations. International cooperation is essential for efficient monkeypox monitoring and control. Countries’ active participation in information sharing, cooperative research projects, and capacity-building efforts can improve the quick exchange of knowledge, resources, and expertise [40]. Such joint actions can strengthen global preparedness and response capabilities, ensuring a united front against the monkeypox threat.

Governments and international organizations must prioritize expenditures in surveillance infrastructure, such as increasing laboratory capacity, improving surveillance systems, and training human resources. Supporting the construction of regional reference laboratories and laboratory networks, as well as providing the resources needed to improve diagnostic capacities in endemic areas, is an important step in effectively monitoring and controlling monkeypox. Along with these initiatives, increasing public knowledge about monkeypox is critical for early case detection and prevention [40]. Educational efforts aimed at informing communities, particularly those at high risk of contracting monkeypox, about the signs, symptoms, and preventive measures connected with this disease are critical. Promoting adequate hygiene practices and encouraging the reporting of suspected cases can assist in early identification and response efforts.

## 6. Conclusions

Monkeypox monitoring necessitates a comprehensive and multidisciplinary approach that includes increased global surveillance, the development of point-of-care diagnostic tools, research on transmission dynamics, advancements in vaccine development and immunization strategies, strengthened international collaboration, investment in surveillance infrastructure, and public awareness. We may successfully monitor and prevent monkeypox outbreaks by actively exploring these pathways, thereby protecting world health.

## Figures and Tables

**Table 1:** Stage, duration, and clinical manifestations of monkeypox infection.

STAGE	DURATION	CHARACTERISTICS
ENANTHEM	0–1 day	The first lesions appear on the tongue and in the mouth.
MACULES	1−2 days	A macular rash appears after the enanthem and spreads from the face to the arms, legs, hands, and feet, including the palms and soles. Within 24 h, the rash usually disseminates and spreads.
PAPULES	1−2 days	On the third day, the rash transforms from macular to papular.
VESICLES	1−2 days	By the fourth to fifth day, lesions can progress to vesicular.
PUSTULES	5−7 day	By day six or seven, pustules form a central depression. After 5–7 days, pustules crust.
SCABS	7−14 days	Pustules crust over by the end of the second week and scabs persist for approximately 7 days before naturally detaching.

**Table 2:** Monkeypoxvirus detection methods and their characteristics.

DETECTION METHOD	DETAILS/REQUIREMENTS	ADVANTAGES	DISADVANTAGE	APPLICATION
REAL-TIME PCR	○ Expanding rapidly ○ Usable across multiple platforms	○ Sensitivity ○ Specificity	○ Cost ○ Technical expertise	➢ Virus detection ➢ Viral load evaluation
MULTIPLEX REALTIME PCR ASSAY	○ PCR method	○ Simultaneous discrimination of clinically similar pathogens	○ Reduced Sensitivity ○ Increased Cost	➢ Virus detection ➢ Viral load evaluation
PAN-ORTHOPOXVIRUS ASSAY	○ PCR method	○ Time-efficient ○ Easy to perform	○ Cross-Reactivity ○ Lack of Target Specificity	➢ Identification of all orthopox genus ➢ Detection in single reaction
LAMP	○ An alternative to RT-PCR ○ No need for complex thremocycler ○ Operates under isothermal condition	○ Time-efficient ○ Easy to perform	○ Primer Design Complexity ○ False-Positive Results	➢ Type identification
RPA	○ Isothermal molecular instrument ○ PCR alternative ○ Complex thremocycler unnecessary	○ Field and point-of-care suitability ○ Rapid and cost-effective ○ Portability	○ Primer Design Complexity ○ Sensitivity to Inhibitors	➢ Virus detection
GENEXPERT	○ Integrated and automated system ○ Work on RT-PCR platform	○ Reduce contamination ○ Appropriate for field conditions	○ Cost ○ Dependency on Cartridges	➢ Virus detection
IMMUNOHISTOCHEMISTRY	○ Histopathological examination	○ Specific detection	○ Sensitivity ○ Specificity	➢ Identification and localization of viral antigens in tissue samples
CELL CULTURE	○ Not used for identification ○ Shows diverse cytopathic effects (CPE) on target cells	○ Vaccine Development Research ○ Viral Pathobiology Studies	○ Subjectivity ○ Limited Sensitivity	➢ Vira isolation and propagation
ELISA	○ Proper for epidemiological studies ○ Practical in endemic regions	○ High Sensitivity ○ Quantitative Results	○ Cross-reactivity	➢ IgM and IgG detection
RAPID ANTIGENIC ASSAY	○ Applicable in endemic regions ○ Suitable for epidemiological studies	○ Quick turnaround time for results	○ Limited Sensitivity ○ Cross-reactivity	➢ Rapid initial screening
GENOME SEQUENCING	○ Suitable for epidemiological studies	precise strain identification and outbreak tracking, but it requires specialized laboratory infrastructure, expertise, and time, cost	○ Cost ○ Data Management and Analysis	➢ Strain identification and tracking of outbreaks

## Data Availability

Not applicable.
